# A Comparative Analysis of Human Behavior Prediction Approaches in Intelligent Environments

**DOI:** 10.3390/s22030701

**Published:** 2022-01-18

**Authors:** Aitor Almeida, Unai Bermejo, Aritz Bilbao, Gorka Azkune, Unai Aguilera, Mikel Emaldi, Fadi Dornaika, Ignacio Arganda-Carreras

**Affiliations:** 1 DeustoTech Institute of Technology, University of Deusto, Av. Universidades 24, 48007 Bilbao, Spain; unai.bermejo@deusto.es (U.B.); aritzbilbao@deusto.es (A.B.); unai.aguilera@deusto.es (U.A.); m.emaldi@deusto.es (M.E.); 2 Department of Computer Science and Artificial Intelligence, University of the Basque Country, M. Lardizabal 1, 20008 Donostia, Spain; gorka.azcune@ehu.eus (G.A.); fadi.dornaika@ehu.eus (F.D.); 3 Ikerbasque, Basque Foundation for Science, Plaza Euskadi, 5, 48009 Bilbao, Spain; ignacio.arganda@ehu.eus; 4 Donostia International Physics Center (DIPC), Manuel Lardizabal 4, 20018 San Sebastian, Spain

**Keywords:** user behavior prediction, behavior modeling, transformers, attention, embeddings, graph neural networks, knowledge graphs, recurrent neural networks, convolutional neural networks, intelligent environments

## Abstract

Behavior modeling has multiple applications in the intelligent environment domain. It has been used in different tasks, such as the stratification of different pathologies, prediction of the user actions and activities, or modeling the energy usage. Specifically, behavior prediction can be used to forecast the future evolution of the users and to identify those behaviors that deviate from the expected conduct. In this paper, we propose the use of embeddings to represent the user actions, and study and compare several behavior prediction approaches. We test multiple model (LSTM, CNNs, GCNs, and transformers) architectures to ascertain the best approach to using embeddings for behavior modeling and also evaluate multiple embedding retrofitting approaches. To do so, we use the Kasteren dataset for intelligent environments, which is one of the most widely used datasets in the areas of activity recognition and behavior modeling.

## 1. Introduction

The advances in hardware and software technologies have led to the adoption of smart-environments in many contexts of our daily life [1]. Environments are equipped with a plethora of devices that can be used to monitor their users. The ability to model and recognize human behavior [2] is essential to create human-centered applications. Behavior modeling has been applied to multiple domains in intelligent environments [3], from home-based healthcare [4] to promoting more sustainable habits [5].

Behavior modeling can follow different approaches in intelligent environments. On the one hand, behavior can be divided into further components (actions, activities, and behaviors) [6], with different levels of abstraction. On the other hand, we can find two different tasks: to identify the behavior or to predict it. Multiple authors have worked on behavior identification, creating algorithms to recognize the performed activities [7]. In this paper, we will focus on the prediction task. Human behavior prediction [8] proves to be necessary in multiple situations. Kuligowski [9] used human behavior prediction to create better evacuation models for fire emergencies. Hatford [10] analyzed the use of deep learning techniques for behavior prediction in strategic settings. In our case, we will focus our prediction on actions on intelligent environments [11]. These are everyday environments enriched with sensor and actuator technologies, which can react and adapt themselves to their users’ needs.

Multiple challenges need to be addressed in order to achieve proper human behavior prediction, both from the machine learning [12] and the intelligent environments [13] perspectives. In our previous research, we proved two beneficial approaches to behavior modeling in intelligent environments: behavior is better represented as a complex structure with different behavior models [14], and representing user actions as embeddings improves the tasks of activity recognition [15] and activity change-point detection [16]. In this paper, we explore the applications of those approaches to the behavior prediction task in intelligent environments. We focus on analyzing the deep learning approaches to human behavior prediction. To do so, we take some of the previously existing state-of-the-art approaches proposed by the authors and compare them to two new approaches: one based on transformers and another based on graph neural networks. All of the proposed methods follow our initial approach of using semantic embeddings to represent the user actions [14], so we also study methods to refine the embedding representation to analyze if it improves the prediction process.

As a result, this paper provides the following contributions.

- A new behavior prediction architecture based on transformers, improving our previous state of the art results.
- An analysis of the feasibility of using graph convolutional networks (GCN) to introduce additional data based on the graph structures inferred from the activities performed.
- An analysis of the usage of retrofitting to improve the embedding representation of the actions.
- A more robust evaluation of the models, providing means and deviation for 100 different executions of the algorithms to better analyze the performance of the models.

The rest of the paper is structured as follows. Section 2 contains an analysis of the state of the art. In Section 3, we describe the embedding-driven architectures we study for behavior modeling. Section 4 contains the performed experiments and the discussion of the results. Finally, in Section 5, we draw the conclusions and propose future areas of research.

## 2. Related Work

User behavior prediction and modeling is a research area which has been applied in several domains using different approaches, such as sensor-based [7] or vision-based [17] techniques. For instance, Deshpande and Deshpande [18] applied behavior prediction for the prediction of online behavior in order to identify malicious users. Irizar et al. [2] discussed how behavior prediction is essential in the creation of energy-efficient and sustainable spaces. Others [19,20] used behavior modeling and prediction for the development of intelligent and automated spaces through Internet of Things (IoT) or even virtual spaces [21]. Troussas et al. [22] applied it to learning analytics for cognitive states and behavior prediction and personalization in order to support learners and further enhance their learning experience. Another relevant domain is pedestrian and vehicle behavior prediction, specially applied to autonomous driving. In [23] authors present MultiPath, which leverages a fixed set of future state-sequence anchors that correspond to modes of the trajectory distribution. At inference, the model predicts a discrete distribution over the anchors and, for each anchor, regresses offsets from anchor waypoints along with uncertainties, yielding a Gaussian mixture at each time step. Jain et al. [24] propose a discrete residual flow network (DRF-NET), a convolutional neural network for human motion prediction that captures the uncertainty inherent in long-range motion forecasting. In particular, the learned network effectively captures multimodal posteriors over future human motion by predicting and updating a discretized distribution over spatial locations. Schulz et al. [25] present a probabilistic prediction framework based on a dynamic Bayesian network, which represents the state of the complete scene including all agents and respects the aforementioned dependencies.They propose Markovian, context-dependent motion models to define the interaction-aware behavior of drivers.

In order to address this issue, several approaches have been used in the literature. More et al. [26] used artificial neural networks to predict and adapt the configuration of a heating controller in intelligent environments. Fatima et al. [27] used support vector machine (SVM) kernel fusion techniques to recognize and predict user activities in smart spaces. Das et al. [28] used sequence matching and compression algorithms together with Markov models to predict users’ mobility patterns. Salian et al. [29] used a saliency detection network augmented with bi-LSTMs (long short-term memory) for behavioral prediction. Thakur and Han [30] propose a framework for human behavior monitoring that aims to take a holistic approach to study, track, monitor, and analyze human behavior during activities of daily living. An LSTM approach was also used in [31] to learn and predict design commands based upon building information modeling (BIM) event log data stored in Autodesk Revit journal files. In [32], authors follow a neuro-fuzzy approach. A Gaussian radial basis function neural network (GRBF-NN) is trained based on the example set generated by a fuzzy rule-based system (FRBS) and the 360-degree feedback of the user. Kim et al. [33] carried out a study using RNN architectures in order to predict multidomain behavior. In the paper, the authors propose the usage of a domain switch-aware holistic recurrent neural network (DS-HRNN) in order to improve the results.

In this manuscript, we introduce a new type of embedding-driven architectures for behavior prediction based on transformers [34] that have improved state-of-the-art results. These architectures, introduced in two of our previous works [14,35], study the usage of two deep neural network approaches such as convolutional neural networks (CNNs) and LSTMs combined with actions represented by neural embeddings. We provide the details of both the previous work architectures and the new ones in Section 3. All of the proposed architectures use a novel conceptual classification for the modeling of user behavior in intelligent environments introduced in [14].

### 2.1. Actions, Activities, and Behaviors

The used conceptual classification represents a user’s behavior in three levels of granularity: actions, activities, and behaviors. As stated in [14], actions could be interpreted as temporally short and conscious muscular movements made by the users, activities are composed of several actions but are temporally longer, and, finally, behaviors describe how the user performs these activities at different times. Moreover, for behaviors, we proposed an extra categorization: intra-activity and inter-activity behaviors. The former one describes how an activity is performed by the user at different times (the actions could be performed in different order but the activity would be the same), whereas the latter describes how the user concatenates different activities in our daily life.

### 2.2. Action Embeddings

The action embedding is an action representation method introduced by us in [14]. This representation method, inspired by Mikolov et al.’s [36] Word2Vec algorithm, which is widely used in natural language processing tasks in order to give semantic meaning to words, allows us to use a rich representation of the actions (the semantic embeddings) in our models, instead of a one-hot-encoding representation where the model does not have any extra information about each of the actions apart from their unique identifier.

The action embeddings are created as follows: given a sequence of actions $S_{act}=\left[a_{1},a_{2},\ldots ,a_{l_{a}}\right]$, where $l_{a}$ is the sequence length and $a_{i}\in {ℜ}^{d_{a}}$ indicates the action vector of the *i*th action in the sequence, we let $Context\left(a_{i}\right)=\left[a_{i-n},\ldots ,a_{i-1},a_{1+1},\ldots ,a_{i+n}\right]$ be the context of the action $a_{i}$, where 2n is the length of the context window. We let $p(a_{i}|Context\left(a_{i}\right))$ be the probability of $a_{i}$ to be in the ith position of the action sequence S. The target of the model used to create the embeddings is to optimize the log maximum likelihood estimation (logMLE):

(1) $$L_{a}\left(MLE\right)=\sum_{a_{i}\in S}\log p\left(a_{i}|Context\left(a_{i}\right)\right)$$

## 3. Proposed Architectures

In this paper, we discuss four embedding-driven architectures for the task of next action prediction. In Section 3.1, we describe the usage of LSTM networks, proposed in our previous work [14]. In Section 3.2, we summarize multiscale convolutional neural networks (MCNNs), proposed in [35]. From these two sections onwards, we start introducing our new contributions. In Section 3.3, we aim to improve our embedding-driven architectures performance with a knowledge-based, graph technique called embedding retrofitting [37]. Similarly, in Section 3.4, we propose graph neural networks (GCNs) as feature extractors for this task. Finally, in Section 3.5, we introduce the transformer architecture to predict user behavior, which outperforms our previous approaches (see Section 4.1). Further details can be found in the implementation (https://github.com/aitoralmeida/DL_HAR, accessed on 13 January 2022) of the models.

### 3.1. Long Short-Term Memory Networks

We presented this architecture in our previous paper [14] focused on predicting users’ behavior. This architecture is a recurrent neural network based on LSTMs that processes the action sequences in three different steps. We divided the model into three sequential modules that process action sequences:

1. Input module: The input module is in charge of receiving the action IDs as one-hot-encodings and transforms them to semantic embeddings. As we demonstrated in [14], using more expressive representations such as embeddings instead of IDs results in better predictions. In the proposed system, and as explained in Section 2.2, the Word2vec algorithm is used to calculate the action embeddings [36], a model widely used in the NLP community. Instead of providing the values directly to the model, we define an embedding layer as the input to the model. In this layer, we store the procedural information on how to transform an action ID to its embedding. This layer trains with the rest of the model. In this way, we can fine-tune the embedding values to the current task, improving the performance of the model [14].
2. LSTM sequence modeling: Receives the action embeddings and is composed of an LSTM layer that is able to capture long short-term dependencies between actions. LSTMs are state-of-the-art recurrent neural networks, a type of network that implements feedback connections to model temporal relations between data points. This module outputs the last action’s features extracted by the LSTM (we do not return the full sequence) and feeds them to the next module.
3. Prediction module: Receives the features extracted by the LSTM and uses those features to predict the next action. This module is composed of two fully connected layers with rectified linear unit (ReLU) activations, dropout, and a final fully connected layer with softmax activation.

For a better understanding of this architecture, refer to Figure 3 in [14]. In that work, we determined, by empirical evaluation, the optimum parameters of the layers, giving the identifier A3 to the best LSTM architecture. For traceability, in this paper, we will refer to that architecture using the same identifier.

### 3.2. Multiscale Convolutional Neural Networks

This architecture is based on our previous work [35]. In a similar fashion to the LSTMs, we divided the action prediction into four modules that process the data sequentially:

1. Input module: The same input module described in the previous section. The input module is in charge of receiving the action IDs as one-hot-encodings and transforms them to semantic embeddings. Instead of providing the values directly to the model, we define an embedding layer as the input to the model. In this layer, we store the procedural information on how to transform an action ID to its embedding. As before, it is configured as trainable.
2. Attention mechanism: Once we have the semantic embeddings for each action, they are processed by the attention mechanism to identify those actions in the sequence that are more relevant for the prediction process. This provides a relevance value for each action. To do so, we use a soft attention mechanism composed of a gated recurrent unit (GRU) encoder, a fully connected layer with a hyperbolic tangent function (tanh) activation, and a fully connected layer with a softmax activation [35]. This approach is similar to how the attention mechanism is applied in NLP to identify the most important words in a sentence, but with a variation regarding the network component to where the attention is applied. Instead of using the attention in the hidden steps of the encoder, we use it in the weights of the action embeddings.
3. MCNN feature extractor: It receives the action embeddings processed by the attention mechanism and is composed of several parallel one-dimensional CNNs, each one analyzing action n-grams of different sizes. These one-dimensional convolution operations are performed actionwise, using the full embedding length for the convolution operation. As we demonstrated in [35], n-grams model relations between actions, which act as useful features to predict the next action in the sequence. These features extracted by the multiple, parallel 1D convolution operations are summarized with pooling operations. After pooling, the parallel branches of the module are merged by means of concatenation. The merged features are flattened before feeding them to the next module.
4. Prediction module: The same prediction module described in the previous section. It receives the features extracted by the MCNN and uses those features to predict the next action. This module is composed of two fully connected layers with rectified linear unit (ReLU) activations, dropout, and a final fully connected layer with softmax activation.

For a better understanding of this architecture, refer to Figure 2 in [35]. Again, by empirical evaluation, in that work we determined the optimum parameters of the layers, giving the identifier M5 to the best MCNN architecture. We tested the same architecture without the attention module. We gave it the identifier M1. For traceability, in this paper, we will refer to that architecture using the same identifier.

### 3.3. Retrofitting Action Embeddings

Retrofitting is a graph-based NLP-original learning technique that employs relational resources to obtain higher quality semantic embeddings [37]. Functionally, it is a post-processing algorithm which runs belief propagation on a knowledge graph to update the original embeddings vectors so that the new ones are similar to the vectors of related embeddings (graph neighbors) and similar to their purely distributional representations.

In the original paper, the authors demonstrated that retrofitting word embeddings with semantic lexicons can improve several NLP tasks such as word similarity, synonym selection, and sentiment analysis [37]. We hypothesize that this technique can also improve the representational capabilities of our action embeddings (as they will be improved with additional information regarding the domain) and, therefore, improve the performance of our embedding-driven architectures in the task of next action prediction. Concretely, we propose to use this method to update the Word2vec action embeddings with three different knowledge graphs (the ones that can be extracted from the dataset) that have the actions as nodes and the relations between actions as edges.

Formally, each of these graphs is an undirected graph G=(V,E) where *V* is a set of unique actions and *E* is a set of edges [x,y] such that x,y∈V. An edge [x,y] is constructed if two actions x,y share a common entity (e.g., the actions “open fridge” and “turn on stove” can be both part of the activity “make dinner”). For the first graph, we construct edges with activities. For the second graph, we construct edges with locations. The third graph has the combination of edges of both the activity and location entities. We believe that introducing both activity and location information in the action embeddings can be useful for predicting user behavior.

Necessarily, these graphs must be generated by experts or from a labeled data source. We provide insights about how the aforementioned graphs can be generated in Section 5. For more information, refer to [37] or the retrofitting software which is publicly available at GitHub/https://github.com/mfaruqui/retrofitting, accessed on 13 January 2022).

### 3.4. Graph Neural Networks

Graph convolutional networks (GCNs) are multilayer neural networks that can directly work on graph structured data [38]. By means of operations called graph convolutions, they generate node embeddings based on the features and properties of their neighbors in a semi-supervised fashion. Namely, each of these node embeddings is a weighted average of its neighbors node features (including itself). Those aggregated node embeddings can later be used as features for several classification tasks, passing them to another neural network for training.

Formally, consider an undirected graph G=(V,E) where *V* is a set of nodes and *E* is a set of edges [39]. In this context, a node is assumed to be connected to itself. Now, a matrix containing all nodes with their features $X\in R^{nxm}$ must be defined, where *n* is the total number of nodes and *m* the dimension of the feature vectors that describe such nodes. Then, $x_{1}\in R^{m}$ is the first node’s feature vector. A symmetric adjacency matrix *A* of G must also be defined, whose diagonal elements are set to 1 because every node is connected to itself (self-loops). Then, the objective of the model, in the first layer, is to generate a new $L^{\left(1\right)}\in R^{nxk}$ node feature matrix that is computed as

(2) $$L^{\left(1\right)}=p\left(\bar{A}XW_{0}\right)$$

where $\bar{A}$ is the symmetric adjacency matrix normalized beforehand, $W_{0}\in R^{mxk}$ is a weight matrix, and *p* is an activation function (e.g., ReLU). Of course, multiple GCN layers can be stacked to capture information about larger neighborhoods [39], generalizing the formula as

(3) $$L^{\left(j+1\right)}=p\left(\bar{A}L^{\left(j\right)}W_{j}\right)$$

where *j* denotes the layer number and $L^{\left(0\right)}=X$.

Curiously, untrained GCNs can serve as feature extractors for nodes in a graph [38]. That is, even with randomly initialized, not-trained weight matrices *W*, they are able to map graph nodes to embeddings with great results. For such reason, we substitute the embedding layer of the aforementioned A3 and M1 architectures with a single, randomly initialized graph convolutional layer that aggregates the features of neighboring action nodes and trains with the rest of the network. We initialize the node feature matrix *X* with the original Word2vec embeddings and construct the adjacency matrix *A* with an undirected graph that has (1) the actions as nodes and (2) the edges when those nodes share a common activity. Our final purpose is to test if the aggregated GCN-based embeddings provide richer information than raw Word2vec action embeddings for next action prediction.

### 3.5. Transformers

The transformer, first introduced by Vaswani et al. [34], is a model architecture based on the use of self-attention mechanism for training and modeling of sequences (in this case, sequences of actions) without using any recurrent approaches such as LSTMs. Although this model has an encoder–decoder architecture, we have only used its encoder. The transformer’s encoder needs positional encodings in order to know the place of each token in the sequence, as no recurrence of any kind is used. Apart from some normalization and feed-forward layers, the encoder has a structure named multi-head attention which is its most important and distinguishable element. The multi-head attention mechanism implements an attention technique named *scaled-dot product attention* [34]. This self-attention method consists of three inputs matrices: queries (Q), which could be defined as the actions to calculate the attention for, keys (K) of dimension $d_{K}$, which are the actions in the sequence with which to compute the attention mechanism, and values (V) of dimension $d_{v}$, where the attention has already been applied. This mechanism can be replicated *h* times (heads) in order to learn different features.

$$Attention\left(Q,K,V\right)=softmax\left(\frac{QK^{T}}{\sqrt{d_{k}}}\right)V$$

**Figure 1:**
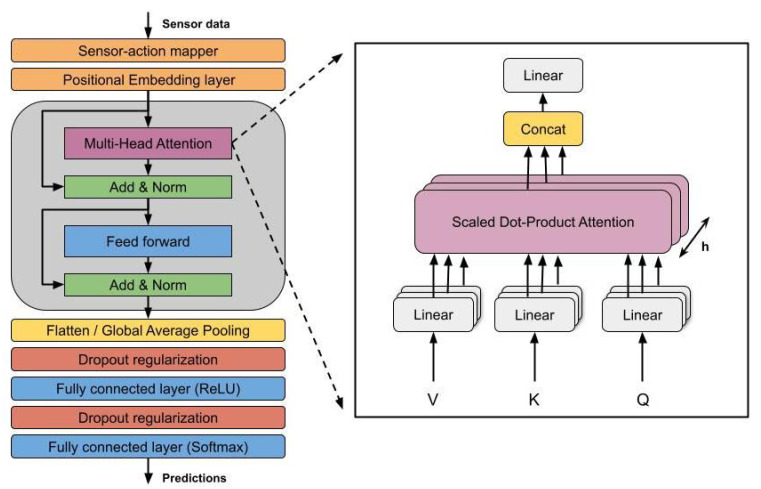
Transformer architecture for behavior modeling.

Therefore, we have used transformer encoders’ input module for the action prediction task, which receives the action IDs and creates its own action embeddings based on the training data. After several tests, we set the number of heads h=3 with a feed-forward size of 300. Regarding the prediction module, we have fed the embeddings to the final fully connected layer using two different approaches: feeding them directly (F1) and using global average pooling (F2). We depict the architecture in Figure 1. This follows the typical transformer architecture, with some modification. First, before the positional embedding layer, we map the sensor data to discrete actions, as in the previous architectures. After this, the actions are processed by the transformer, which extracts the features that define the action sequence. This feature extraction process is learned during the training. Finally, we classify those features using two fully connected layers.

## 4. Evaluation

To evaluate the proposed architectures, we have used the Kasteren dataset [40]. This dataset is a well-known, public benchmark used in the activity recognition literature, which permits other researchers to compare their approaches with the results of our work.

The dataset is the outcome of monitoring a 26-year old man in a three-room apartment with 14 binary sensors. Those sensors were installed in locations such as doors, cupboards, toilet, or freezer. Sensor data for 28 days was collected for a total of 2120 sensor events and 245 activity instances. The annotated activities were: ‘LeaveHouse’, ‘UseToilet’, ‘TakeShower’, ‘GoToBed’, ‘PrepareBreakfast’, ‘PrepareDinner’, and ‘GetDrink’. As in our previous works [14,35], the sensor events were mapped one to one to actions, resulting in the following set of actions: ‘UseDishwasher’, ‘OpenPansCupboard’, ‘ToiletFlush’, ‘UseHallBedroomDoor’, ‘OpenPlatesCupboard’, ‘OpenCupsCupboard’, ‘OpenFridge’, ‘UseMicrowave’, ‘UseHallBathroomDoor’, ‘UseWashingmachine’, ‘UseHallToiletDoor’, ‘OpenFreezer’, ‘OpenGroceriesCupboard’, and ‘UseFrontdoor’. For making the evaluation process more streamlined, we applied this sensor-action mapping offline. A more in-depth discussion of the dataset can be found in [15].

**Figure 2:**
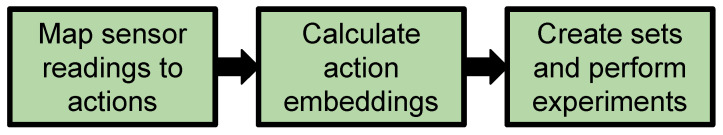
Evaluation steps.

In order to prepare the experiments, the steps (see Figure 2) were the following:

1. The sensor readings are mapped to specific actions as previously discussed in [15]. This results in discrete action, e.g., ‘UseDishwasher’, ‘OpenPansCupboard’, ‘ToiletFlush’, etc. The ‘None’ activity present in the Kasteren dataset is maintained.
2. The embeddings of those actions are calculated using the Word2Vec algorithm as discussed in [14]. This results in an embedding of 50 dimensions associated with each discrete action.
3. After transforming the actions to embeddings, the validation is performed in the manner described below.

For the validation, the dataset was split into a training set (80%) and a validation set (20%) of continuous days. To carry out the training, we use *n* actions as the input to predict the next action. That is, the task is modeled as a supervised learning problem where the training examples are the sequences of actions and the label is the next action that follows each sequence. We found n=5 as the optimum value for predicting the next action in the sequence length’s experiments of our very first work [14]. Hence, we fix this value of *n* for the new architecture’s experiments of this paper.

The proposed architectures (see Table 1) were implemented with Keras [41] and executed using TensorFlow [42] as the backend in a Quadro RTX 8000 GPU. We include our best previous approaches A3, M1, and M5 to compare them to the ones proposed in this paper. Similarly, for the sake of completeness, we do also include a variant of the A3 architecture that employs the embedding-level attention mechanism [35] identified as A10.

All the models were trained for 1000 epochs, with a batch size of 128, using categorical cross entropy as the loss function and Adamax [43] as the optimizer. Adamax is a variant of Adam based on the infinity norm, most of the times more stable and suitable for embeddings than Adam. After the 1000 epochs, we selected the best model using the validation accuracy as the fitness metric. The action embeddings were calculated using the Word2vec [36] algorithm on its Gensim implementation. We selected an embedding size of 50 float values. The embedding layer of all architectures was configured as trainable. Notice the transformers do not use those Word2vec embeddings.

Regarding the knowledge graphs for GCNs and retrofitting, we employed two different sources of information to generate them: expert activity models (EAMs) and the labeled data of the Kasteren dataset. In [15], we defined an EAM as a standardized computational model of activities generated by experts that contains information about (1) the minimum number of actions to perform the activity, (2) the activity duration, (3) the activity starting time ranges, and (4) the location where the activity is usually performed. With this knowledge, it is possible to determine which actions share activities and locations to construct edges. Likewise, we propose to generate edges from Kasteren’s labeled data. This could be a perfect alternative when expert knowledge is not available. We do simply construct an edge if two actions share a common label (entity) at any time in the dataset. For example, an edge between ’UseHallBathroomDoor’ action and ’ToiletFlush’ action could be constructed as they share the ’UseToilet’ activity (the label) at time 9:14:01 and time 9:14:06, respectively.

Following the same strategy as in our previous papers [14,35], we selected top-k accuracy as the evaluation metric, which can be defined as

(4) $$acc\_at\_k=\frac{1}{N}\sum_{i=1}^{N}b\left[a_{i}\in C_{i}^{k}\right]$$

where $a_{i}$ is the ground-truth action and $C_{i}^{k}$ is the set of the top *k* predicted actions. We define the scoring function as b[.]→{0,1}. When the condition in the first part is true, the function value is 1. Otherwise, the value is 0. That is, if the ground-truth action is in the set of the top *k* predicted actions, the function returns 1. Otherwise, the function returns 0. We provide the accuracy for *k* values of 1, 2, 3, 4, and 5.

Finally, due to the stochastic nature of the models and GPU parallelism, we execute each architecture 100 times, reporting average and standard deviation values. This allows us to make a more robust evaluation by reducing the randomness bias, and, hence, extract better conclusions and make our experiments more reproducible.

### Results and Discussion

We report the architecture experiments results in Table 2. For accuracy at 1, we can clearly see how the transformers F1 and F2 outperform our previous architectures both in mean and maximum values. For accuracy at 2, transformers do also provide a good average performance, tied with M5. For the rest of *k* values, A3 is, on average, the best architecture. Therefore, one of the conclusions that can be drawn from these results is that, for sequences of actions, self-attention mechanisms such as multi-head attention perform better than recurrent approaches (LSTMs). Namely, transformers learn relationships between actions with multi-head attention mechanisms and positional embeddings, avoiding long dependency issues that recurrent networks suffer when maintaining a state vector between time steps. These state vectors that try to model such temporal dependencies between actions do not exist in the transformer architecture. Transformers use an attention layer that can access all the previous actions, weighting them according to a learned measure of relevancy. This eliminates the necessity of maintaining a representation of previous actions with a state vector, which has more information and is more difficult to keep the longer the sequence is. Transformers also perform better than the CNN-based approaches, providing an improved accuracy. This results are in line with the comparisons performed in other domains between LSTM-, CNN-, and transformer-based architectures. Results in the natural language processing (NLP) domain provide the same conclusions in different NLP tasks. As we base our action representations in embeddings (a similar approach is used in NLP to represent words), it is expected that the most competitive models in one domain also outperform other models in intelligent environments. The obtained results show that transformer-based models are more suited to behavior prediction than the ones based on CNNs [14,35] or recurrent neural networks [14,29,31,32,33,35].

Regarding the poor performance of GCNs, we believe that it is related to all individual action information being lost during the graph convolution (node feature aggregation). Intuitively, actions which share activities are getting very similar embeddings, losing their individual, distributional features captured by the Word2vec algorithm. This is probably impacting our embedding-driven architectures that need to distinguish between actions to model the next action prediction problem. Due to these results, we encourage researchers not to employ GCNs as feature extractors for this task. Regardless of the results for the action prediction task, we would expect that the GCNs would perform better at activity level, using the action embeddings to calculate the aggregated one for the activity. We think that the recent advances in geometric deep learning could be applied to different behavior modeling tasks in intelligent environments, specifically to activity recognition and to change point detection.

We report the retrofitting experiments results in Table 3. First, we can see how our best LSTM approach A3 obtains average improvements in accuracy at 1, 2, and 5. It also achieves the best maximum accuracy at 1 when performing retrofitting. The activity and location information seems useful to predict the next action for this architecture. For A10, our LSTM with embedding-level attention, the results are even better. Retrofitting improves the average values of all k. A10 seems to take advantage of the location and activity information introduced into the action embeddings. Nevertheless, it is clear that there exist differences between graphs, which indicates that the source of information and the chosen entity to generate edges is important. We believe that the appropriate graph must be determined empirically and is scenario-dependent. Second, the results confirm that for M1, our MCNN without attention, retrofitting can be useful, improving the mean values of accuracy at 1, 4, and 5, but with smaller differences. For our M5 approach that employs embedding-level attention, no average improvements are achieved (only in peak accuracy at 1). We conclude that LSTMs are more capable of managing the activity and location information than MCNNs, which rely on learning action n-grams, rather than modeling long short-term dependencies between them. Namely, MCNNs learn relevant relations between actions to predict user behavior, for which location and activity information is probably not as useful. Furthermore, these retrofitting experiments confirm that, although the embeddings are fine-tuned to the specific task (the embedding layer is configured as trainable), their original quality (viz. representational capabilities) has a direct impact on the proposed models.

Last, but not least, we would like to remark on the superiority of the transformer architecture, especially at the hardest evaluation, accuracy at 1, in comparison to the rest of the proposed architectures (with and without retrofitting). To the best of our knowledge and until new novel generalist architectures appear, we believe that we are in front of the performance upper bound for this dataset, as transformers are known to be state-of-the-art models in other fields (especially in NLP).

## 5. Conclusions and Future Work

In this paper, we analyzed multiple deep learning approaches to human behavior prediction in intelligent environments. We compared our previous state-of-the-art results with more recent techniques in a robust evaluation, providing summary metrics. We showed that transformer-based approaches provide better results than approaches based on CNNs (such as the ones used in [14,35]) or recurrent neural networks (used in [14,29,31,32,33,35]). The GCN approach did not achieve the expected performance, mainly due to how the used graphs were constructed. While we discourage the use of GCNs in the action prediction tasks following the same approach to construct the graphs, we believe that the recent advances in geometric deep learning are applicable to the behavior modeling domain, as discussed in the future work. We also studied how to improve the action embeddings in order to achieve better results. The obtained results show that transformers offer the best performance for the task, and that in those models that use embeddings, fine-tuning them with retrofitting also improves the results.

As future work, we plan on continuing analyzing the feasibility of GCNs for action prediction, studying how to build proper knowledge graphs that would allow us to exploit previous knowledge. Concretely, we believe that we should model the problem with dynamic directed graphs, constructing weighted edges between actions depending on how they co-occur in the time series. We think that these graphs will allow us to extract better features after the graph convolution. We do also believe that introducing temporal features after the graph convolution is a key issue, as other researchers have studied before [44] and as we have tried in our G1 architecture. Another research area that we want to explore is the addition of interpretability mechanisms to the action prediction model, in order to make the results more explainable. Finally, another aspect that we would like to address is the use of light models that can be run in embedded hardware. One of the challenges in intelligent environments is being able to distribute the intelligence in the IoT infrastructure [13]. We would like to study models such as MobileNet or EfficientNet and compare their performance to the models presented in this paper.

## Figures and Tables

**Table 1:** Proposed architectures. ID is the architecture identifier. Description is a brief explanation of the underlying model. Attention describes the type of attention mechanism used in the model. Dense # is the number of fully connected layers with a ReLU activation (all the architectures have a final fully connected layer with a softmax activation). Dense size is the size of the fully connected layers with an ReLU activation. Dropout is the value of the dropout regularizations.

ID	Description	Attention	Dense #	Dense Size	Dropout
A3 [14]	Standard LSTM size 512	None	2	1024	0.8
A10	Standard LSTM size 512	Embedding-level	2	1024	0.8
M1 [35]	Multi-scale CNN N-grams: 2, 3, 4, 5	None	2	512	0.8
M5 [35]	Multi-scale CNN N-grams: 2, 3, 4, 5	Embedding-level	2	512	0.8
G1	GCN size 50 step num 1 and A3	None	2	1024	0.8
G2	GCN size 50 step num 1 and M1	None	2	512	0.8
F1	Transformer with flatten	Multi-head	1	800	0.8
F2	Transformer with GAP	Multi-head	1	800	0.8

**Table 2:** Architecture experiments. Accuracy at *k*. Mean accuracy and standard deviation for 100 executions. Maximum accuracy at 1 is also reported. ID is the architecture identifier.

ID	Mean *k* = 1	Mean *k* = 2	Mean *k* = 3	Mean *k* = 4	Mean *k* = 5	Max *k* = 1
A3	0.4388 ± 0.0077	0.6064 ± 0.0189	**0.7324** ± 0.0146	**0.7982** ± 0.0065	**0.8513** ± 0.0069	0.4573
A10	0.4253 ± 0.0060	0.6038 ± 0.0098	0.6863 ± 0.0127	0.7802 ± 0.0123	0.8318 ± 0.0096	0.4487
M1	0.4197 ± 0.0090	0.5938 ± 0.0121	0.6941 ± 0.0109	0.7583 ± 0.0089	0.8052 ± 0.0090	0.4530
M5	0.4387 ± 0.0125	**0.6240** ± 0.0146	0.7074 ± 0.0163	0.7925 ± 0.0119	0.8321 ± 0.0107	0.4701
G1	0.2150 ± 0.0143	0.3164 ± 0.0199	0.4150 ± 0.0424	0.5397 ± 0.0440	0.6274 ± 0.0348	0.2479
G2	0.2092 ± 0.0093	0.3214 ± 0.0066	0.4331 ± 0.0420	0.5627 ± 0.0303	0.6469 ± 0.0298	0.2479
F1	**0.4600** ± 0.0097	0.6237 ± 0.0182	0.7190 ± 0.0153	0.7748 ± 0.0148	0.8294 ± 0.0126	**0.4915**
F2	0.4575 ± 0.0091	0.6233 ± 0.0199	0.7092 ± 0.0112	0.7883 ± 0.0120	0.8405 ± 0.0139	0.4829

**Table 3:** Retrofitting experiments with different knowledge graphs. Accuracy at *k*. Mean accuracy for 100 executions. Maximum accuracy at 1 is also reported. ID is the architecture identifier. Source is the information from which the knowledge graph was generated. EAM: expert activity model, Dataset: information from Kasteren et al.’s manuscript [40] and the labeled data. Graph indicates the entity that was used to generate edges. ACT: activities, LOC: locations, ACT-LOC: activities and locations.

ID	Source	Graph	Mean *k* = 1	Mean *k* = 2	Mean *k* = 3	Mean *k* = 4	Mean *k* = 5	Max *k* = 1
A3	None	None	0.4388	0.6064	**0.7324**	**0.7982**	0.8513	0.4573
	EAMs	ACT	0.4345	0.6080	0.7096	0.7872	0.8492	0.4573
		LOC	0.4342	0.6094	0.7139	0.7877	0.8495	0.4530
		ACT-LOC	0.4361	0.6108	0.7154	0.7895	0.8495	0.4573
	Dataset	ACT	0.4329	0.6178	0.7189	0.7902	**0.8517**	0.4573
		LOC	0.4368	0.6112	0.7142	0.7880	0.8491	0.4615
		ACT-LOC	**0.4439**	**0.6207**	0.7199	0.7888	0.8476	**0.4701**
A10	None	None	0.4253	0.6038	0.6863	0.7802	0.8318	0.4487
	EAMs	ACT	0.4329	0.6195	0.6988	0.7938	0.8330	0.4573
		LOC	0.4325	0.6198	0.6993	**0.7959**	**0.8342**	0.4658
		ACT-LOC	0.4317	0.6190	0.6978	0.7944	0.8335	0.4487
	Dataset	ACT	0.4353	**0.6229**	0.6995	0.7926	0.8304	**0.4701**
		LOC	0.4338	0.6215	0.7002	0.7953	0.8329	0.4573
		ACT-LOC	**0.4363**	0.6195	**0.7011**	0.7922	0.8259	0.4615
M1	None	None	0.4197	**0.5938**	**0.6941**	0.7583	0.8052	**0.4530**
	EAMs	ACT	0.4163	0.5899	0.6790	0.7559	0.8133	0.4359
		LOC	0.4182	0.5895	0.6816	0.7570	0.8134	0.4402
		ACT-LOC	0.4174	0.5892	0.6803	0.7553	0.8148	0.4359
	Dataset	ACT	0.4148	0.5884	0.6839	0.7578	0.8140	0.4402
		LOC	**0.4203**	0.5892	0.6822	0.7587	0.8150	0.4487
		ACT-LOC	0.4186	0.5892	0.6862	**0.7648**	**0.8179**	0.4402
M5	None	None	**0.4387**	**0.6240**	**0.7074**	**0.7925**	**0.8321**	0.4701
	EAMs	ACT	0.4059	0.5799	0.6717	0.7493	0.8053	0.4615
		LOC	0.4123	0.5863	0.6757	0.7534	0.8065	0.4701
		ACT-LOC	0.4090	0.5815	0.6747	0.7550	0.8083	0.4615
	Dataset	ACT	0.4022	0.5756	0.6696	0.7489	0.8051	0.4615
		LOC	0.4121	0.5808	0.6768	0.7552	0.8060	0.4658
		ACT-LOC	0.4125	0.5876	0.6813	0.7589	0.8076	**0.4744**

## References

[1] Sánchez-Corcuera R., Nuñez-Marcos A., Sesma-Solance J., Bilbao-Jayo A., Mulero R., Zulaika U., Azkune G., Almeida A. (2019). Smart cities survey: Technologies, application domains and challenges for the cities of the future. Int. J. Distrib. Sens. Netw..

[2] Irizar-Arrieta A., Gómez-Carmona O., Bilbao-Jayo A., Casado-Mansilla D., Lopez-De-Ipina D., Almeida A. (2020). Addressing behavioural technologies through the human factor: A review. IEEE Access.

[3] Ranasinghe S., Al Machot F., Mayr H.C. (2016). A review on applications of activity recognition systems with regard to performance and evaluation. Int. J. Distrib. Sens. Netw..

[4] Bennett J., Rokas O., Chen L. (2017). Healthcare in the smart home: A study of past, present and future. Sustainability.

[5] Irizar-Arrieta A., Casado-Mansilla D., Retegi A., Laschke M., López-de-Ipiña D. (2020). Exploring the Application of the FOX Model to Foster Pro-Environmental Behaviours in Smart Environments. Sensors.

[6] Chaaraoui A.A., Climent-Pérez P., Flórez-Revuelta F. (2012). A review on vision techniques applied to human behaviour analysis for ambient-assisted living. Expert Syst. Appl..

[7] Chen L., Hoey J., Nugent C.D., Cook D.J., Yu Z. (2012). Sensor-based activity recognition. IEEE Trans. Syst. Man Cybern. Part.

[8] Cook D.J., Krishnan N.C. (2015). Activity Learning: Discovering, Recognizing, and Predicting Human Behavior from Sensor Data.

[9] Kuligowski E. (2013). Predicting human behavior during fires. Fire Technol..

[10] Hartford J.S. (2016). Deep Learning for Predicting Human Strategic Behavior. Ph.D. Dissertation.

[11] Brumitt B., Meyers B., Krumm J., Kern A., Shafer S. (2000). Easyliving: Technologies for intelligent environments. International Symposium on Handheld and Ubiquitous Computing.

[12] Subrahmanian V.S., Kumar S. (2017). Predicting human behavior: The next frontiers. Science.

[13] Patrono L., Atzori L., Šolić P., Mongiello M., Almeida A. (2020). Challenges to be addressed to realize Internet of Things solutions for smart environments. Future Gener. Comput. Syst..

[14] Almeida A., Azkune G. (2018). Predicting human behaviour with recurrent neural networks. Appl. Sci..

[15] Azkune G., Almeida A. (2018). A scalable hybrid activity recognition approach for intelligent environments. IEEE Access.

[16] Bermejo U., Almeida A., Bilbao-Jayo A., Azkune G. (2021). Embedding-based real-time change point detection with application to activity segmentation in smart home time series data. Expert Syst. Appl..

[17] Weinl D., Ronfard R., Boyer E. (2011). A survey of vision-based methods for action representation, segmentation and recognition. Comput. Vis. Image Underst..

[18] Deshpande D., Deshpande S. (2017). Analysis of various characteristics of online user behavior models. Int. J. Comput. Appl..

[19] Mulero R., Almeida A., Azkune G., Abril-Jiménez P., Waldmeyer M.T., Castrillo M.P., Patrono L., Rametta P., Sergi I. (2018). An IoT-aware approach for elderly-friendly cities. IEEE Access.

[20] Zhan Y., Haddadi H. (2019). Towards automating smart homes: Contextual and temporal dynamics of activity prediction. Adjunct Proceedings of the 2019 ACM International Joint Conference on Pervasive and Ubiquitous Computing and Proceedings of the 2019 ACM International Symposium on Wearable Computers.

[21] Köse A., Tepljakov A., Petlenkov E. Dynamic predictive modeling approach of user behavior in virtual reality based application. Proceedings of the 2019 27th Mediterranean Conference on Control and Automation (MED).

[22] Troussas C., Krouska A., Virvou M. (2020). Using a multi module model for learning analytics to predict learners’ cognitive states and provide tailored learning pathways and assessment. Machine Learning Paradigms.

[23] Chai Y., Sapp B., Bansal M., Anguelov D. (2019). Multipath: Multiple probabilistic anchor trajectory hypotheses for behavior prediction. arXiv.

[24] Jain A., Casas S., Liao R., Xiong Y., Feng S., Segal S., Urtasun R. Discrete residual flow for probabilistic pedestrian behavior prediction. Proceedings of the Conference on Robot Learning, PMLR.

[25] Schulz J., Hubmann C., Löchner J., Burschka D. Interaction-aware probabilistic behavior prediction in urban environments. Proceedings of the 2018 IEEE/RSJ International Conference on Intelligent Robots and Systems (IROS).

[26] Morel N., Bauer M., El-Khoury M., Krauss J. (2001). Neurobat, a predictive and adaptive heating control system using artificial neural networks. Int. J. Sol. Energy.

[27] Fatima I., Fahim M., Lee Y.K., Lee S. (2013). A unified framework for activity recognition-based behavior analysis and action prediction in smart homes. Sensors.

[28] Das S.K., Cook D.J., Battacharya A., Heierman E.O., Lin T.Y. (2002). The role of prediction algorithms in the MavHome smart home architecture. IEEE Wirel. Commun..

[29] Salian N. (2018). Visual Attention and Memory Augmented Activity Recognition and Behavioral Prediction. Proceedings of the International Conference on Applications and Techniques in Information Security.

[30] Thakur N., Han C.Y. (2021). An Ambient Intelligence-Based Human Behavior Monitoring Framework for Ubiquitous Environments. Information.

[31] Pan Y., Zhang L., Skibniewski M.J. (2020). Clustering of designers based on building information modeling event logs. Comput. Civ. Infrastruct. Eng..

[32] Dash C.S.K., Behera A.K., Dehuri S., Cho S.B. (2020). Building a novel classifier based on teaching learning based optimization and radial basis function neural networks for non-imputed database with irrelevant features. Appl. Comput. Inform..

[33] Kim D., Kim S., Zhao H., Li S., Rossi R.A., Koh E. Domain switch-aware holistic recurrent neural network for modeling multi-domain user behavior. Proceedings of the Twelfth ACM International Conference on Web Search and Data Mining.

[34] Vaswani A., Shazeer N., Parmar N., Uszkoreit J., Jones L., Gomez A.N., Kaiser Ł., Polosukhin I. (2017). Attention is all you need. Advances in Neural Information Processing Systems.

[35] Almeida A., Azkune G., Bilbao A. Embedding-level attention and multi-scale convolutional neural networks for behaviour modelling. Proceedings of the 2018 IEEE SmartWorld, Ubiquitous Intelligence & Computing, Advanced & Trusted Computing, Scalable Computing & Communications, Cloud & Big Data Computing, Internet of People and Smart City Innovation (SmartWorldSCALCOMUICATCCBDComIOPSCI).

[36] Mikolov T., Sutskever I., Chen K., Corrado G.S., Dean J. (2013). Distributed representations of words and phrases and their compositionality. Advances in Neural Information Processing Systems.

[37] Faruqui M., Dodge J., Jauhar S.K., Dyer C., Hovy E., Smith N.A. (2014). Retrofitting word vectors to semantic lexicons. arXiv.

[38] Kipf T.N., Welling M. (2016). Semi-supervised classification with graph convolutional networks. arXiv.

[39] Yao L., Mao C., Luo Y. Graph convolutional networks for text classification. Proceedings of the AAAI Conference on Artificial Intelligence.

[40] Van Kasteren T., Noulas A., Englebienne G., Kröse B. Accurate activity recognition in a home setting. Proceedings of the 10th International Conference on Ubiquitous Computing.

[41] Chollet F. (2015). Keras. GitHub. https://github.com/fchollet/keras.

[42] Abadi M., Barham P., Chen J., Chen Z., Davis A., Dean J., Devin M., Ghemawat S., Irving G., Isard M. Tensorflow: A system for large-scale machine learning. Proceedings of the 12th USENIX Symposium on Operating Systems Design and Implementation (OSDI 16).

[43] Kingma D.P., Ba J. (2014). Adam: A method for stochastic optimization. arXiv.

[44] Zhao L., Song Y., Zhang C., Liu Y., Wang P., Lin T., Deng M., Li H. (2019). T-gcn: A temporal graph convolutional network for traffic prediction. IEEE Trans. Intell. Transp. Syst..

[45] Biewald L. (2020). Experiment Tracking with Weights and Biases. https://www.wandb.com/.

